# Cortical thickness and emotion processing in young adults with mild to moderate depression: a preliminary study

**DOI:** 10.1186/s12888-016-0750-8

**Published:** 2016-02-24

**Authors:** Bernice A. Fonseka, Natalia Jaworska, Allegra Courtright, Frank P. MacMaster, Glenda M. MacQueen

**Affiliations:** Mathison Centre for Mental Health Research & Education, Department of Psychiatry; Hotchkiss Brain Institute (HBI), University of Calgary, 7th Floor, Teaching, Research & Wellness (TRW) Building, 3280 Hospital Drive NW, Calgary, AB T2N 4Z6 Canada; Department of Psychiatry, McGill University, Montreal, QC Canada; Child and Adolescent Imaging Research (CAIR) Program; Alberta Children’s Hospital Research Institute, University of Calgary, Calgary, AB Canada; Department of Paediatrics, University of Calgary, Calgary, AB Canada; Strategic Clinical Network for Addictions and Mental Health, Alberta Health Services, Calgary, Canada

**Keywords:** Major depressive disorder, Cortical thickness, Emotion processing, Adolescents/young adults

## Abstract

**Background:**

Major depressive disorder (MDD) is a multifaceted illness involving cognitive, emotional, and structural brain changes; illness onset typically occurs in adolescence or young adulthood. Cortical thickness modulations may underlie, or accompany, functional brain activity changes in the prefrontal cortex (PFC) during emotional processing that tend to be observed in MDD.

**Methods:**

Thirteen unmedicated young adults with mild to moderate MDD, aged 18–24, completed a facial expression Go/No Go task and underwent a magnetic resonance imaging (MRI) scan to assess cortical thickness. Cortical thickness and performance on the Go/No Go task was also assessed in age-matched healthy comparison subjects (HCs; *N* = 14).

**Results:**

Participants with depression had thicker left pars opercularis cortices than HCs. They also exhibited impaired response inhibition to neutral faces when responding only to sad faces, and a faster response time overall.

**Conclusions:**

Though our sample size is limited, this pilot study nevertheless provides evidence for cortical thickening in left frontal brain regions in a non-severely depressed, young adult group compared to healthy controls. There was also evidence of disturbances in emotion processing in this group.

## Background

Major depressive disorder (MDD) is a prevalent and multi-faceted illness involving both cognitive and emotional symptoms, among others [1]. Illness onset typically occurs during adolescence and early adulthood [2], and psychiatric morbidity during this critical neurodevelopmental period can adversely impact education, occupational outcomes, and relationships [3]. From a treatment and prevention perspective, understanding MDD in its early stages is highly valuable; however, the neurobiological and behavioural profile of the disorder in younger populations is not well characterized. Two features of interest are cortical thickness, a brain morphometric measure, and emotion processing, which is highly relevant to the cognitive and social aspects of MDD.

### Cortical thickness & MDD

Across all ages, potential cortical thickness modulations in MDD are relatively under-explored despite the possibility that these could serve as diagnostic markers, or as targets for therapeutic intervention. Much of the existing literature on cortical thickness has focused on late-life MDD (>60 years of age). These studies generally show no differences between MDD and healthy control groups (e.g., [4, 5]), or cortical thinning in frontal and para/postcentral regions as well as the cuneus/isthmus in MDD patients [6–8].

A handful of studies have assessed cortical thickness patterns in depressed non-elderly adults (<60 years of age). For example, one study found cortical thickness reductions in the dorsal and ventrolateral prefrontal cortices (PFC) in actively depressed subjects, compared to those in remission and controls [9]. Unexpectedly, remitted patients had increased thickness in several regions compared to controls, including the pregenual and subgenual anterior cingulate cortices, the anterior PFC, right inferior parietal lobule, and superior temporal gyrus. This could represent a trait marker related to remission, or reflect the consequences of effective pharmacotherapy. Another group found that individuals with both early- (before 25) and late-onset MDD showed cortical thinning in the dorsolateral PFC (DLPFC) compared to non-depressed individuals [10]. However, the early-onset group also demonstrated thickening in some regions (posterior cingulate and fusiform gyrus), and thinning in others (parahippocampal gyri) when compared to the late-onset group. Jaworska et al. [11] reported thickening of the frontal poles in early onset MDD patients (before 24). In yet another study, Han et al. [12] reported no differences in cortical thickness between first episode MDD participants and controls, although a volumetric reduction of the caudal anterior cingulate gyrus existed in patients. Overall, the spatial patterns of cortical thickness alterations in both elderly and non-elderly depressed adults remain unclear and factors such as childhood trauma, medication use, and age of disorder onset may play modulating roles.

Even less research has focused on cortical thickness in depressed children and adolescents. One study found that depressed children had reduced cortical thickness in parietal and temporal regions, yet increased thickness in the temporal poles [13]. Given the involvement of frontal regions/PFC in MDD [14], and based on some of the existing adult literature, Reynolds et al. [15] hypothesized that adolescents with MDD would show cortical thinning in the middle frontal gyrus (part of the DLPFC), but instead found thickening in this region and the anterior cingulate. In contrast, another study found that young adults with a family history of mood disorders had reduced cortical thickness in the right parahippocampal and fusiform gyrus [16]. As such, these conflicting results warrant further examination of cortical thickness in younger depressed cohorts, in whom the effects of prolonged disease burden and medication use can be minimized.

### Emotion processing in MDD

Depression is associated with reduced accuracy in facial expression identification (e.g., [17]). People with depression have been shown to exhibit a memory bias and enhanced neural processing for sad faces and/or interpret neutral faces more negatively than non-depressed individuals [18–20]. Disturbances in emotion processing circuits involving cortico-limbic connections are generally thought to underlie these behavioural abnormalities [21].

One paradigm used to examine cognitive control of emotion processing is the emotive Go/No Go task. Typically, participants respond to certain facial expressions (“Go” trials) embedded among infrequent non-target facial expressions (“No Go” trials). This assesses an individual’s ability to recognize and respond to goal-relevant emotive stimuli, while withholding preponent responses to non-target expressions.

In a version of the task where participants responded to emotive words, two research groups showed that medicated and unmedicated depressed patients exhibited an attentional bias toward sad stimuli as reflected by faster response times to sad versus happy targets [22, 23]. A subsequent facial expression Go/No Go study found comparable results, wherein depressed children and adolescents had faster reaction times to sad faces than controls [24]. However, a second facial expression Go/No Go study of depressed adolescents/young adults failed to find any reaction time differences compared with non-depressed controls, though faster reaction times to emotive faces in the MDD cohort were associated with greater depression symptom severity [25].

Although the neural processes associated with the Go/No Go task are not well understood, response selection to non-emotive Go, and inhibition to No Go, stimuli has been associated with activity in the pre-supplementary motor area [26]. Additionally, Elliot et al. [27] reported that the inferior frontal gyrus and anterior/subgenual cingulate cortex are engaged when responding to targets of differing emotional valence. Emotion processing can be modulated by treatment since performance on an affective Go/No Go task improved following repetitive transcranial magnetic stimulation (rTMS) of the DLPFC in depressed patients [28]. As such, the Go/No Go task appears to involve largely frontal regions; though attention and visual networks likely play a large role in task execution.

Few studies have assessed the relation between cortical thickness and performance on the emotive Go/No Go task, or comparable tasks. Nevertheless, one meta-analysis found that greater PFC cortical thickness was associated with better executive functioning [29], indicating a link between cognition and this metric. In a group of healthy older adults, lateral parietal thickness was positively associated with visuomotor speed and set shifting ability [30], which are important features of the Go/No Go task. Given that the Go/No Go task invokes preponent response inhibition, it is interesting to note that cortical thickness, particularly in the frontal regions (left middle and superior frontal gyrus, orbitofrontal cortex), has been inversely associated with impulsiveness in healthy adults [31] and adolescents [32]. Mak et al. [33] found that anterior cingulate and inferior orbitofrontal cortex gray matter reductions in depressed females was correlated with poorer performance on an emotion regulation task. These data suggest that attention, inhibition, and emotion processing may be related to cortical thickness, particularly in MDD. Assessing older adolescents and young adults with depression is particularly useful for isolating the early stages of the disease, as younger patients are less likely to have an extensive disease burden or medication load. This study therefore investigated cortical thickness and emotion processing using the facial expression Go/No Go task in young adults with and without MDD. We hypothesized that compared to healthy controls, young adults with MDD would show cortical thickness disruptions in PFC regions, as well as impaired response inhibition to emotive stimuli on a facial expression Go/No Go task.

## Methods

### Participants

Study participants were young adults (18–24 years old) with MDD (*N* = 13) and non-depressed healthy controls (HCs, *N* = 14). Following an initial telephone screen, potentially eligible participants were invited for an in-person clinical assessment (Mini International Neuropsychiatric Interview [MINI]; [34]). The Hamilton Rating Scale for Depression (HAMD_17_; [35]) was administered to determine depression severity; eligible MDD participants had a minimum score of 14 (moderate severity). Those with scores of 8–13 (mild severity) or those with scores of >19 (severe MDD) were considered on a case-by-case basis. A primary MDD diagnosis was confirmed by the study psychiatrist (G.M.). All MDD patients were untreated (i.e., not taking antidepressant drugs or engaged in formal psychotherapy) at the time of testing (testing commenced after >1 month wash-out for previously-medicated participants). Patients with severe depression were not actively recruited as they were more likely to be on some kind of antidepressant intervention, and it would have been unethical to ask them to stop. Seven patients had previously taken antidepressant medication for their MDD; the number of previous major depressive episodes (MDE) ranged from 0 to 9 (average: 3.1 lifetime episodes, including current one), and age of first MDE onset ranged from 9 to 22 years of age (average: 15.3 years).

Notable exclusion criteria were: history of bipolar disorder, psychosis, anorexia/bulimia or seizures; current substance abuse; and significant suicide risk. Written informed consent was obtained prior to study initiation and this study was approved by the Conjoint Health Research Ethics Board at the University of Calgary. As part of the informed consent process, participants agreed to the publication of the aggregated and anonomized data that was collected.

### Facial expression Go/No Go task

**Fig. 1:**
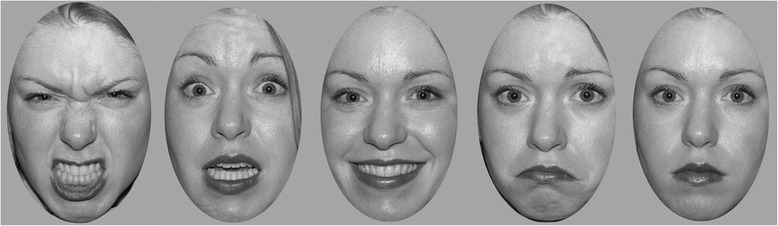
Sample faces used in the facial expression Go/No Go task. Left to right: anger, fear, happy, sad and neutral expressions

In our facial expression Go/No Go task [24], participants responded to frequent “Go” trials while withholding their preponent response to infrequent “No Go” trials. The stimuli consisted of faces depicting four emotions (anger, fear, happy, sad) and neutral expressions (adapted from the NimStim Face Stimulus Set; http://www.macbrain.org/resources.htm). Faces were black and white photographs of young adult males and females (equal proportion; various ethnicities), not wearing glasses or makeup. The neck, hair, and ears were excluded (i.e., images were presented in an oval-shaped mask; Fig. 1).

Stimuli were presented on a PC laptop using ePrime software (Psychology Software Tools, Pittsburgh, PA), and participants either had to respond (via a keyboard press) to a target emotion and not respond to neutral faces, or vice versa, yielding eight conditions (Anger/Fear/Happy/Sad Go & Neutral No Go; Neutral Go & Anger/Fear/Happy/Sad No Go). In this paper, these conditions are referred to by the emotion only (e.g., Anger Go or Anger No Go). For each condition, there were 30 Go trials and 10 No Go trials (40 trials total). Faces were presented for 500 ms, followed by an inter-trial interval consisting of a variable length fixation cross, presented in the middle of the screen (900–1200 ms). The percentage of correct hits, false alarms (FA; response to a No Go trial) and mean response time (RT; ms) to hits were assessed for each of the eight conditions.

### Structural MRI acquisition and cortical thickness analyses

All neuroimaging was carried out with a General Electric Discovery 750 W 3 T scanner with a 24-channel head coil. A high-resolution T1-weighted anatomical scan in the axial orientation was obtained using the following parameters: repetition time (TR) = 8160 ms, time to echo (TE) = 3.16 ms, flip angle = 10°, 300 × 300 matrix, field of view (FOV) = 240 mm, 226 axial slices, and 0.8 mm slice thickness.

Cortical thickness analysis was conducted using FreeSurfer software (http://surfer.nmr.mgh.harvard.edu). Briefly, brain images were corrected for intensity and contrast irregularities due to magnetic field inhomogeneities [36]. After an automated skull-stripping procedure, images were manually edited to remove remaining extra-cerebral voxels. In the segmentation step, the gray and white matter interface was determined based on intensity and geometric information [36]. A triangular surface tessellation was then applied to each hemisphere, and smoothed using a deformable surface algorithm [36]. The surface of each image was inflated to better visualize cortical folding patterns and sulci [37]. The inflated cortical surface was then transformed into a parameterizable surface [37] and aligned with a reference brain template [38]. Cortical thickness was calculated by taking the smallest distance between the pial surface and gray-white matter interface. FreeSurfer parcellates each hemisphere into 34 regions and derives an average thickness (mm) for each of these [39].

### Statistical analyses

Group differences (MDD, HC) in HAMD_17_ scores, age, and years of education were assessed using student’s t-tests. Sex ratio was assessed using a Chi-square analysis.

We divided our Go/No Go task analyses into two parts. The first dealt with “Emotion Go” conditions where emotional faces (anger, fear, happy or sad) were the Go and neutral faces the No Go conditions. The second dealt with “Emotion No Go” conditions where neutral faces were the Go trials and emotional faces the No Go trials. Repeated-measures analyses of variance (rmANOVAs; *p* < .05) were conducted for hits, FAs, and RT with group (MDD, HC) as the between-subjects factor and emotion as the within-subjects factors for each of the “Emotion Go” and “Emotion No Go” analyses. Significance was set at *p* < .01 for all post hoc tests.

Cortical thicknesses were imported as average mm values from FreeSurfer into the Statistical Package for the Social Sciences (SPSS) software for Macintosh, Version 20 (IBM, Armonk, NY), which was used to carry out all statistical analyses. Student’s t-tests were used to assess for group differences in each of the 34 regions per hemisphere (*p* < .01). Exploratory Spearman’s correlations (*p* < .001) were performed between cortical thickness of regions that tended to be (*p* < .05-.01) or were different (*p* < .01) between groups, and continuous clinical/demographic characteristics (i.e., age, education, HAMD_17_ score) and Go/No Go outcomes.

## Results

### Participant characteristics

Demographics and clinical characteristics are presented in Table 1. One HC did not complete the emotive task. No group differences (MDD vs. HCs) existed in terms of sex ratio, age or years of education. As expected, MDD patients had higher HAMD_17_ scores than HCs [*t*(25) *=* 9.89, *p* < .001]. The main outcome measures for the affective Go/No Go task are presented in Table 2. Pairwise comparisons, used to follow-up significant main effects of emotion, are presented in Table 3.

**Table 1:** Demographics and clinical characteristics of depressed (MDD) and healthy control (HC) groups

	MDD (*N* = 13)	HC (*N* = 14)
Gender (M/F)	7/6	7/7
Age (yr)	21.5 ± 1.5	21.0 ± 1.8
Education (yr)	14.5 ± 1.7	15.0 ± 1.5
HAMD_17_ score***	15.3 ± 5.0	1.4 ± 1.6
Age of onset (yr)	15.6 ± 3.9	--
Time since diagnosis (yr)	5.9 ± 3.8	--
Ethnicity		
% Caucasian	84.6	50.0
% Asian	15.4	21.4
% East Asian	--	21.4
% Middle Eastern	--	7.1

Means ± standard deviation presented. ***p < .001

HAMD_17_: Hamilton Depression Rating Scale (17 item version)

**Table 2:** Main outcomes from the Go/No Go task for all eight conditions by diagnostic group

	Mean Hits (%)		Mean FA (%)		Mean RT (ms)	
Condition	MDD (*N* = 13)	HC (*N* = 13)	MDD (*N* = 13)	HC (*N* = 13)	MDD (*N* = 13)	HC (*N* = 13)
Anger Go	96.6 ± 1.5	91.5 ± 17.2	20.5 ± 9.6	12.6 ± 7.0	378.0 ± 42.6	443.4 ± 118.6
Fear Go	95.8 ± 9.6	95.4 ± 10.1	10.8 ± 10.0	4.6 ± 5.2	411.0 ± 43.3	466.8 ± 100.7
Happy Go	98.9 ± 3.9	98.0 ± 3.5	9.2 ± 7.9	6.9 ± 9.5	379.0 ± 39.6	429.3 ± 111.9
Sad Go	95.6 ± 4.6	93.3 ± 7.2	47.5 ± 11.4	28.5 ± 12.8	436.4 ± 32.3	475.0 ± 106.6
Anger No Go	97.5 ± 5.3	93.3 ± 8.6	14.2 ± 6.7	11.5 ± 3.8	402.1 ± 40.6	474.0 ± 105.1
Fear No Go	93.9 ± 11.4	86.9 ± 13.2	13.3 ± 13.0	3.1 ± 4.8	419.7 ± 50.5	521.6 ± 140.1
Happy No Go	95.6 ± 8.6	80.3 ± 19.7	13.3 ± 13.0	3.1 ± 4.8	427.0 ± 67.7	484.1 ± 82.6
Sad No Go	89.7 ± 7.5	86.4 ± 11.1	44.2 ± 15.1	32.3 ± 14.8	450.3 ± 53.8	509.1 ± 93.9
All conditions	95.4 ± 6.3	90.6 ± 6.3	21.6 ± 6.3	13.1 ± 6.3	412.9 ± 67.0	475.4 ± 67.0

Means ± standard deviation presented

*MDD* depressed group, *HC* healthy control group, *FA* false alarms, *RT* response time

**Table 3:** Pairwise comparisons between emotions from the Go/No Go task

		Result	Statistic^a^
Emotion Go Conditions	Hits	--	--
	FA	More FA in Sad Go vs. all other conditions	min *p* < .001
		More FA in Anger Go vs. Fear & Happy Go conditions	*p* < .001; *p* = .003, respectively
	RT	Faster RT for Anger vs. Fear Go condition	*p* = .007
		Faster RT for Happy vs. Sad Go condition	*p* = .003
Emotion No Go Conditions	Hits	More hits in Anger No Go vs. all other conditions	min *p* = .004
	FA	More FA in Sad No Go vs. all other conditions	min *p* < .001
		More FA in Anger No Go vs. Happy No Go condition	*p* = .014 (trend)
	RT	Faster RT for Anger No Go vs. Fear & Sad No Go conditions	*p* < .001; *p* = .011, respectively

*FA* false alarms, *RT* response time

^a^This statistical comparison represents follow-up tests of a repeated measures ANOVA (significant main effect of emotion)

### Go/No Go task performance

No main effects or interactions existed on hits in the Emotion Go analysis. However, there was a main effect of emotion on hits in the Emotion No Go analysis [*F*(3,72) = 6.10, *p* = .001; Table 3]. Additionally, the MDD group tended to exhibit more hits than HCs in the Emotion No Go analysis [*F*(1,24) = 3.98, *p* = .06]. Assessment of the significant emotion × group interaction [*F*(3,72) = 3.85, *p* = .01] did not reveal any significant group differences, although MDD patients tended to have more hits to neutral targets than HCs in the Happy No Go condition (*p* = .02).

**Fig. 2:**
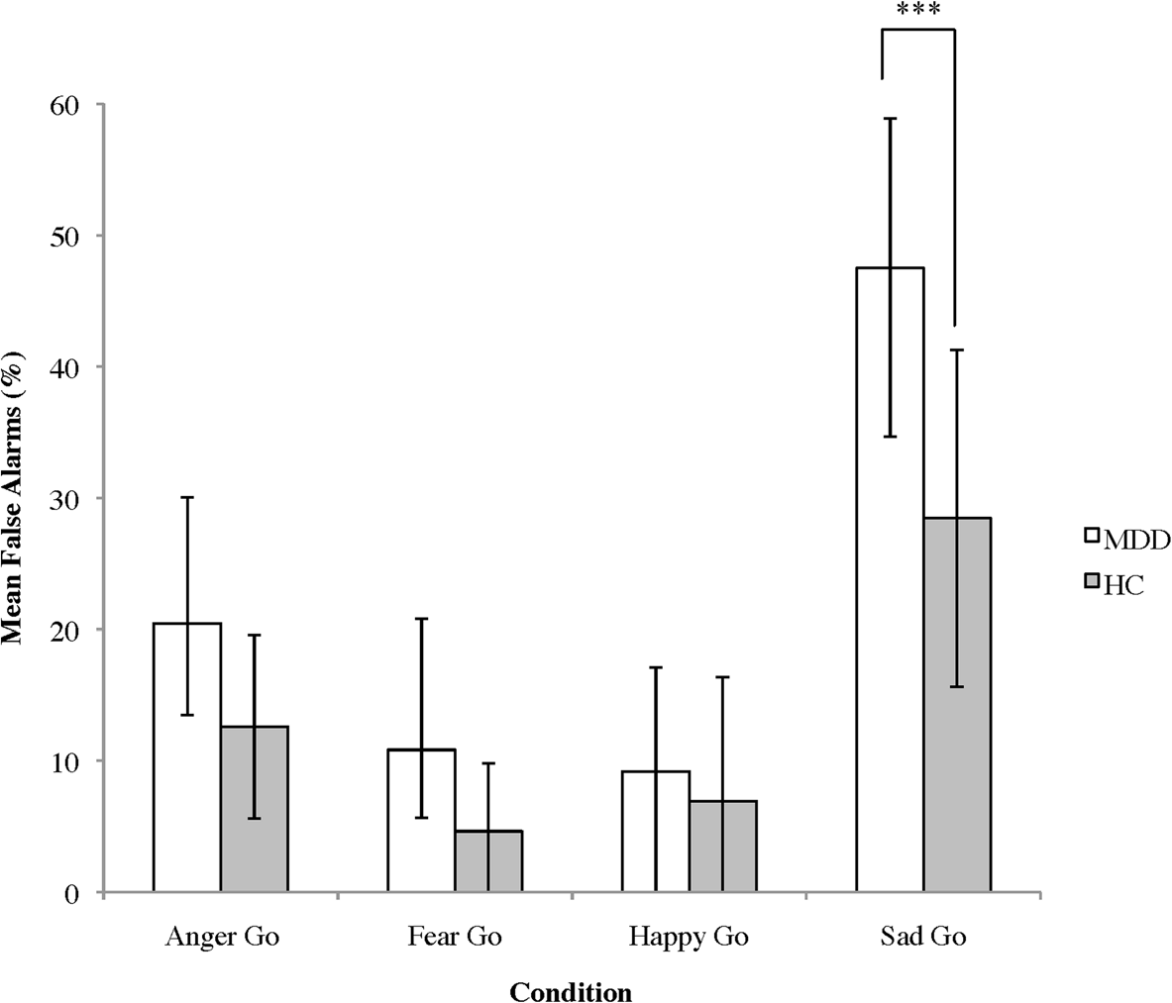
False alarms from the facial expression Go/No Go task in emotion Go conditions. MDD: depressed group, HC: healthy control group. Values indicate the mean, error bars represent standard deviation, stars indicate significance (***p < .001)

In terms of FAs, a main effect of emotion existed for the Emotion Go analysis [*F*(3,69) = 67.74, *p* < .001; Table 3]. A main effect of group was also found [*F*(1,23) = 14.74, *p* = .001], with patients having more FAs than HCs (i.e., responses to neutral faces). Follow-up of the significant emotion × group interaction [*F*(3,69) = 4.35, *p* = .007] indicated that MDD patients had more FAs than HCs in the Sad Go condition (*p* < .001; Fig. 2). Similarly, there were main effects of both emotion [*F*(3, 72) = 59.72, *p* < .001] and group [*F*(1, 24) = 7.44, *p* = .01] on FAs in Emotion No Go conditions (Table 3). MDD patients had nearly twice the number of Emotion No Go FAs as HCs, but no emotion × group interaction existed.

There was a main effect of emotion on RT in both the Emotion Go [*F*(3,69) = 4.02, *p* = .03] and Emotion No Go [*F*(3,72) = 3.76, *p* = .03] analyses (Table 3). Patients tended to have faster RTs than HCs [*F*(1,23) = 4.00, *p* = .06] in Emotion Go conditions; this was significant in the Emotion No Go analysis [*F*(1,24) = 6.65, *p* = .02]. No group × emotion interactions existed.

### Cortical thickness

The MDD group had a thicker left pars opercularis compared to HCs, while eight other regions tended to exhibit greater cortical thickness in MDD patients [*t*(24) = 3.12, *p* = .005; Table 4]. Exploratory Spearman correlations were carried out between cortical thickness in the nine regions listed in Table 4 with Go/No Go outcomes. These were conducted across groups to maximize our ability to detect significant relations. Left pars opercularis cortex thickness was positively related to FAs in the Sad Go [ρ(23) = .72, *p* < .001] and Sad No Go conditions [ρ(23) = .63, *p* = .001]. Similarly, the left supramarginal cortex thickness was positively correlated with FAs in the Sad Go condition [ρ(23) = .61, *p* = .001]. When split by group, none of these were significant at *p* < .001. Similar correlations between cortical thickness and continuous clinical/demographic characteristics yielded no significant results.

**Table 4:** Select regional cortical thickness values

Region	Mean thickness (mm)		Group comparison
	MDD (*N* = 13)	HC (*N* = 14)	
LH pars opercularis	2.61 ± .10	2.49 ± .10	*t*(24) = 3.12, *p* = .005
LH pars orbitalis	2.77 ± .18	2.60 ± .19	*t*(24) = 2.38, *p* = .026
LH pars triangularis	2.51 ± .11	2.41 ± .12	*t*(24) = 2.33, *p* = .029
LH precuneus	2.45 ± .80	2.38 ± .06	*t*(24) = 2.61, *p* = .015
LH rostral middle frontal	2.42 ± .09	2.32 ± .10	*t*(24) = 2.63, *p* = .015
LH supramarginal	2.52 ± .11	2.43 ± .08	*t*(24) = 2.41, *p* = .024
RH fusiform	2.76 ± .17	2.64 ± .11	*t*(24) = 2.30, *p* = .032
RH pars opercularis	2.62 ± .13	2.50 ± .11	*t*(24) = 2.49, *p* = .020
RH posterior cingulate	2.77 ± .10	2.65 ± .14	*t*(24) = 2.51, *p* = .020

*LH* left hemisphere, *RH* right hemisphere, *MDD* depressed group, *HC* healthy control group

Means ± standard deviation presented.

## Discussion

This pilot study investigated emotion processing and cortical thickness in young adults with MDD compared with non-depressed controls. Group differences in performance on the facial expression Go/No Go task were noted, particularly in FAs and RT. Patients tended to have thicker cortices in a number of brain regions. Exploratory assessments yielded a handful of significant correlations between cortical thickness and Go/No Go performance outcome measures. However, these results have to be interpreted cautiously, given our small sample size. Nevertheless, there is some indication that cortical thickness in the left frontal and parietal lobes were related to response inhibition under conditions involving sad stimuli.

### Emotion processing

Depressed participants demonstrated a speed-accuracy trade-off in that they responded faster to emotional stimuli but had more FAs, especially in the Sad Go condition, than healthy controls. The faster RT across conditions is unintuitive given that psychomotor retardation is a feature of depression [40]. Our findings are consistent, however, with previous work showing that faster RT in the facial expression Go/No Go task was associated with depression in adolescents [24] and young adults [25]. Han et al. [25] speculated that faster RTs may be a product of emotional reactivity and reduced executive control. In support of this, Hare et al. [41] showed that emotional reactivity in adolescence (e.g., impaired response inhibition to emotive No Go stimuli) is related to enhanced amygdala activity combined with reduced top-down PFC regulation.

Increased FAs in depressed participants in the Sad Go condition is consistent with findings that MDD patients tend to interpret neutral faces more negatively than controls [42]; in other words, they tend to exhibit a negative cognitive bias [43]. However, another Go/No Go study in adult females revealed that depressed participants also had impaired response inhibition to *non*-emotive stimuli (letters); this was attributed to compromised inhibitory control [17]. As such, disrupted executive/inhibitory control may have played some role in the elevated FAs evident in this study, and sad stimuli may have led to further perturbation of executive function, or induced a greater interaction between emotive and cognitive control centers. These explanations are supported by an event-related potential [electroencephalography (EEG)-derived] study suggesting that healthy controls actively attend away from sad faces, while MDD patients fail to display the same inhibitory processes [44]. Other neuroimaging work has shown that depressed subjects have increased activation of the left parahippocampal gyrus and left amygdala in response to sad faces, compared with healthy individuals [18]. Biased emotional processing and altered inhibitory control may also contribute to some of the emotional and social features of the illness [45].

Across diagnostic groups, angry expressions seemed to elicit similar behavioural responses as sad expressions in terms of FAs. However, all participants generally responded to angry faces more rapidly than to sad ones. This increased emotional reactivity may be related to the evolutionary and ecological value of angry faces as threatening stimuli [46].

### Cortical thickness

Participants with depression had a thickening of the left pars opercularis (orbital aspect of the inferior frontal gyrus) compared to HCs. A number of other left hemisphere structures, including the adjacent pars orbitalis and pars triangularis, and DLPFC regions also tended to be thicker in the MDD group. This is consistent with one study that reported thicker cortices in the nearby frontal poles of depressed adults with pediatric onset MDD [11]. The structural and functional maturation of the frontal lobe continues into early adulthood [47]; as such, our participant cohort was likely in a stage of active cortical development. Longitudinal imaging has previously shown that cortical thickness in different brain regions follow different developmental curves – for example, thickness in most of the lateral frontal cortex peaks at adolescence, declines briefly, and stabilizes in adulthood [48]. It is possible that the thicker cortices in specific brain regions in MDD participants reflect a developmental trajectory that is modulated by the presence of depression. These changes could also represent a neurocompensatory response to MDD or perhaps result from previous treatment (though, at testing, participants were treatment-naïve).

Interestingly, the left inferior frontal gyrus has been implicated in response inhibition [49]; abnormalities in this region in MDD patients may be related to the heightened reactivity observed in the facial expression Go/No Go task. However, it is difficult to draw direct relations between structural disturbances and cognitive performance, as our behavioural task likely recruited multiple brain regions and complex processing systems [41, 50, 51].

### Limitations and future directions

Several study limitations should be acknowledged. The primary weakness is the relatively limited sample size, which also prevented the investigation of putative sex effects. In terms of the facial expression Go/No Go task, our experiment did not explicitly investigate the functional correlates of emotion processing. Future studies should involve a functional imaging component to better understand how emotion processing differs in young adults with MDD. Further, assessing the influence of depression severity on cortical thickness and emotional processing in a young adult cohort is warranted in comparable future work. Additionally, possible confounding effects of past medication or psychotherapy were not controlled for, and individuals in the age range we tested are in an active stage of brain development. These issues may have increased variability and made it especially difficult to detect any group differences in cortical thickness.

## Conclusion

In summary, we examined emotion processing and cortical thickness in young adults with MDD. There was evidence for cortical thickening in left frontal brain regions in the MDD group, and a number of behavioral differences compared to HCs on a facial expression Go/No Go task, namely faster RTs and impaired response inhibition in MDD particularly in the context of sad expressions. Our results may reflect interactions of MDD with brain development in young adults, or represent a neurocompensatory response to MDD that occurs early in the disease.

The young adult age group has been largely under-explored with regard to depression, and this paper helps to characterize the neurobiological and behavioural profile of the disorder in younger populations.

## Abbreviations

DLPFC: dorsolateral prefrontal cortex
EEG: electroencephalography
FA: false alarm
FOV: field of view
HAMD_17_: hamilton rating scale for depression (17-item version)
HC: healthy control
MDD: major depressive disorder
MDE: major depressive episode
MINI: Mini International Neuropsychiatric Interview
MRI: magnetic resonance imaging
PFC: prefrontal cortex
rmANOVA: repeated measures analysis of variance
RT: response time
rTMS: repetitive transcranial magnetic stimulation
SPSS: statistical Package for the Social Sciences
TE: time to echo
TR: repetition time
